# In-operando control of sum-frequency generation in tip-enhanced nanocavities

**DOI:** 10.1038/s41377-025-01855-5

**Published:** 2025-05-22

**Authors:** Philippe Roelli, Isabel Pascual Robledo, Iris Niehues, Javier Aizpurua, Rainer Hillenbrand

**Affiliations:** 1 CIC nanoGUNE BRTA, 20018 Donostia-San Sebastián, Spain; 2 Materials Physics Center, CSIC-UPV/EHU, 20018 Donostia-San Sebastián, Spain; 3 Institute of Physics, University of Münster, 48149 Münster, Germany; 4 Donostia International Physics Center (DIPC), 20018 Donostia-San Sebastián, Spain; 5 IKERBASQUE, Basque Foundation for Science, 48013 Bilbao, Spain; 6 Department of Electricity and Electronics, University of the Basque Country (UPV/EHU), 48940 Leioa, Spain

**Keywords:** Nanophotonics and plasmonics, Nonlinear optics, Nanocavities, Infrared spectroscopy

## Abstract

Sum-frequency generation (SFG) is a second-order nonlinear process widely used for characterizing surfaces and interfaces with monolayer sensitivity. Recently, optical field enhancement in plasmonic nanocavities has enabled SFG with continuous wave (CW) lasers from nanoscale areas of molecules, promising applications like nanoscale SFG spectroscopy and coherent upconversion for mid-infrared detection at visible frequencies. Here, we demonstrate CW SFG from individual nanoparticle-on-mirror (NPoM) cavities, which are resonant at visible frequencies and filled with a monolayer of molecules, when placed beneath a metal scanning probe tip. The tip acts as an efficient broadband antenna, focusing incident CW infrared illumination onto the nanocavity. The cascaded near-field enhancement within the NPoM nanocavity yields nonlinear optical responses across a broad range of infrared frequencies, achieving SFG enhancements of up to 14 orders of magnitude. Further, nanomechanical positioning of the tip allows for in-operando control of SFG by tuning the local field enhancement rather than the illumination intensities. The versatility of tip-enhanced nanocavities allows for SFG studies of a wide range of molecular species in the few-molecule regime without the need for complex nanofabrication. Our results also promise SFG nanoimaging with tips providing strong visible and IR field enhancement at their apex, offering a robust platform for future applications in nonlinear nanooptics.

## Abstract (web summary)

We demonstrate on-demand CW sum-frequency generation from nanoparticle-on-mirror cavities positioned beneath a scanning probe tip. The tip controls visible and infrared fields within the nanocavity, allowing for tunable nonlinear spectroscopy.

## Graphical abstract

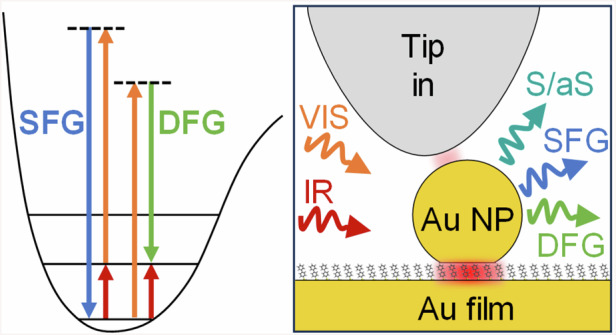

## Introduction

Sum-frequency generation (SFG) is a coherent second-order nonlinear process^1–3^ with significant applications in up-conversion-based and vibration-selective imaging and spectroscopy^4–7^. The SFG process is particularly effective for probing vibrational modes at interfaces where the material second-order nonlinearity, *χ*^(2)^, is often activated. By carefully tuning the infrared (IR) frequency to the vibrational mode *ν* and phase matching IR and visible (VIS) input beams, a coherent SFG output beam at frequency *ω*_SFG_ = *ω*_IR_(*ν*) + *ω*_VIS_ can be detected, revealing subtle details about the molecular environment and bonding at the interface.

Compared to coherent anti-stokes Raman spectroscopy (CARS), vibrational spectroscopy via SFG offers the advantage of requiring only a single Raman process, the second Raman process being replaced by a resonant IR process. This results in SFG having significantly higher intrinsic cross-sections than CARS. However, both techniques suffer from diffraction limitations, hindering nanoscale spatial resolution and the observation of few or even single molecules. CARS overcomes this limitation using plasmonic nanoparticles or scanning probe tips, which confine and enhance fields, enabling enhanced sensitivity and spatial resolution for sensing and imaging^8,9^. Additionally, surface- and tip-enhanced CARS enables time-resolved monitoring of vibrations reaching the single molecule level^10,11^.

Surprisingly, tip-enhanced SFG^12^ remains a widely unexplored terrain. Only recently, cavity-enhanced SFG has been implemented^13^ in self-assemblies of organic molecules with the help of nanoparticle-on-mirror (NPoM) cavities^14^, in which the incident VIS and IR fields are concentrated in a 1 nm high and a few 10 nm wide gap between a particle and a mirror, defined by the thickness of the molecular layer and the facet of the particle, respectively. When the VIS-resonant NPoM cavity is combined with an IR-resonant antenna in a carefully engineered way, the infrared field enhancement in the NPoM gap can be strongly boosted. In this case, remarkable SFG and difference frequency generation (DFG) signals can be observed simultaneously even with low power continuous wave (CW) illumination, opening a new path for efficient up-conversion and detection of IR photons with visible cameras^15^.

So far, efficient cavity-enhanced SFG relies on sophisticated fabrication of doubly resonant cavities and may require design variation for the study of different molecular vibrational modes. Importantly, the spatial overlap of IR and VIS fields inside the gap has to be well-defined to maximize signal yield and to avoid spurious cavity responses that may prevent the observation of clear SFG signals^16^. Further, because of the relatively inefficient coupling between far-field radiation and plasmonic nanocavities, the enhanced SFG signal fails to approach the up-conversion efficiencies of optomechanical devices operating in different frequency ranges^17^. For all these reasons, a more versatile and active platform allowing for the adjustment of spectral and spatial characteristics of the cavity is desirable, for example, to push SFG spectroscopy towards single molecule sensing and for evaluating a large number of molecular species for future IR to VIS up-conversion applications.

Here we demonstrate efficient CW infrared to visible SFG and DFG in NPoM nanocavities filled with a monolayer of organic molecules, which is enhanced and controlled in-operando with the metal tip of a scattering-type scanning near-field optical microscope (s-SNOM). The tip acts as an antenna in both IR and VIS spectral ranges, concentrating the incident fields of both frequencies at its apex. The apex fields serve as a local illumination for the NPoM cavity, enhancing the fields in the gap between the particle and mirror. By recording second-order nonlinear responses (SFG and DFG) under infrared illumination at two distinct frequencies, separated by 500 cm^−1^ and both tuned to specific molecular vibrational modes, we demonstrate efficient coherent upconversion across the mid-infrared spectral range. Key to this achievement is the non-resonant but strong IR field concentration at the tip apex of the s-SNOM. Numerical simulations of the relevant field enhancements verify our experimental results and let us explain the remarkable upconversion signals through a cascaded near-field enhancement, similar to that of an efficient nanolens made of a chain of metal nanoantennas^18,19^, and through momentum conservation facilitated by the broad momentum distribution of the near fields^20^ in the NPoM nanocavities. Our study not only highlights cascaded enhancements of optical signals—reporting the nonlinear response of molecular vibrations—by up to 14 orders of magnitude but also introduces a method for actively controlling field enhancements at visible and infrared frequencies through precise 3D nanomechanical positioning of the tip.

## Results

**Fig. 1:**
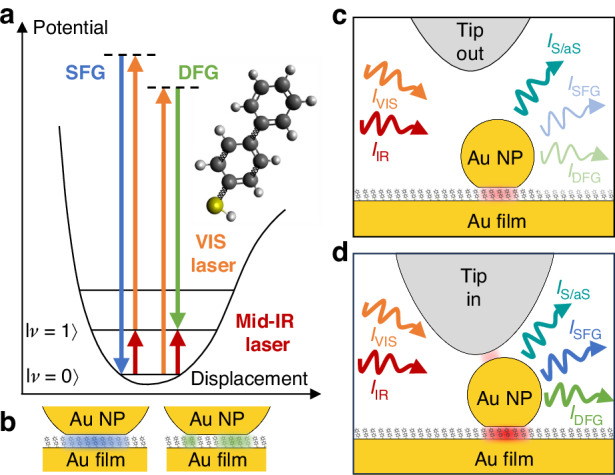
Nonlinear optics inside a tip-enhanced NPoM cavity. **a** Jablonski energy diagram of coherent upconversion signals for one vibrational mode of the BPT molecule (inset). **b** Illustration of the field distributions inside the BPT functionalized NPoM gap at the frequencies where SFG (blue) and DFG (green) occur. **c**, **d** Illustration of vibrational signals from a tip-controlled NPoM cavity in two configurations: tip out of contact (**c**), and tip in contact (**d**) with the NPoM. The molecules in the NPoM cavity, under both infrared (*I*_IR_) and visible (*I*_VIS_) illuminations, generate Stokes/anti-Stokes (S/aS), SFG and DFG vibrational signals

The concept of tip-controlled nonlinear optics based on a molecules-filled NPoM cavity is outlined in Fig. 1. We first illustrate in Fig. 1a the intrinsic vibrational SFG and DFG processes of an organic molecule using a Jablonski energy diagram. When molecules are simultaneously illuminated by visible light (orange arrow) and by a IR radiation (red arrow) tuned to a molecular vibrational mode that is both Raman and IR active, i.e. *χ*^(2)^ active^3^, the targeted vibrational mode mediates the generation of SFG and DFG signals at the angular frequencies *ω*_+_ = *ω*_VIS_ + *ω*_IR_ (upper vibrational sideband) and *ω*_−_ = *ω*_VIS_ − *ω*_IR_ (lower vibrational sideband), respectively. During SFG, the vibrational mode is brought to its first excited state by IR photons (red arrow in in Fig. 1a) and transitions via Raman scattering to its ground state (blue arrow). In contrast, during DFG, the vibrational mode is simultaneously excited Raman scattering (green arrow) and IR photons.

To enhance vibrational optical processes, we embed organic molecules into NPoM cavities, that is, into the gap between a gold nanoparticle and a gold film (illustrated in Fig. 1b). Under VIS illumination alone, the Stokes (S) and anti-Stokes (aS) signals intensities, *I*_S/aS_ (cyan arrow in Fig. 1c, d), are enhanced by factors ${{\mathcal{F}}}_{{\rm{S}}}$ and ${{\mathcal{F}}}_{{\rm{aS}}}$, which are given by^21^:

1 $${{\mathcal{F}}}_{{\rm{S/aS}}}={F}_{{\rm{VIS}}}\,{F}_{{\rm{-/+}}}$$

where *F*_VIS_, *F*_+_ and *F*_−_ are the position-dependent near-field intensity enhancement factors inside the NPoM gap at the angular frequencies *ω*_VIS_, *ω*_+_ and *ω*_−_ (surface-enhanced Raman scattering, SERS). Under additional IR illumination, the signal enhancement factors of vibrational SFG and DFG (light blue and green arrows) for a molecule at a specific location in the gap can be described by^13^:

2 $${{\mathcal{F}}}_{{\rm{SFG}}}={F}_{{\rm{IR}}}\,{F}_{{\rm{VIS}}}\,{F}_{+}$$

3 $${{\mathcal{F}}}_{{\rm{DFG}}}={F}_{{\rm{IR}}}\,{F}_{{\rm{VIS}}}\,{F}_{-}$$

where *F*_IR_ is the position-dependent near-field intensity enhancement factor inside the NPoM gap at the angular frequency *ω*_IR_. Due to the absence of both plasmonic and geometric resonances in small Au particles at IR frequencies, *F*_IR_ remains weak. Consequently, SFG and DFG signals of intensity ${I}_{{\rm{SFG}}/{\rm{DFG}}}\propto {\int}_{{{\rm{V}}}_{{\rm{mol}}}}{{\mathcal{F}}}_{{\rm{SFG}}/{\rm{DFG}}}{\rm{dV}}$^22^, where V_mol_ indicates the volume occupied by molecules, are too small to be detected with the use of an NPoM cavity. Note that we omit potential phase and collective vibrational effects, as their contributions within a cavity remain debated^23,24^. To overcome the problem of weak nonlinear signals, we place a nanomechanically controlled metal tip above the NPoM cavity (Fig. 1b, c). The tip primarily serves as a broadband non-resonant infrared antenna, concentrating the incident IR field at its apex. In close proximity to the Au nanoparticle (Fig. 1c), the near field at the tip apex provides an additional illumination of the NPoM cavity, increasing substantially *F*_IR_ and thus the SFG and DFG signal intensities. We note that under visible and infrared CW illuminations both anti-Stokes (*I*_aS_, linear) and SFG (*I*_SFG_, nonlinear) signals appear simultaneously at *ω*_+_, whereas *I*_S_ and *I*_DFG_ overlap at *ω*_−_. In general, the signals appearing on these upper and lower vibrational sidebands, ${I}_{+}={I}_{{\rm{aS}}}+{I}_{{\rm{SFG}}}={c}_{{\rm{aS}}}\,{{\mathcal{F}}}_{{\rm{aS}}}+{c}_{{\rm{SFG}}}\,{{\mathcal{F}}}_{{\rm{SFG}}}$ and ${I}_{-}={I}_{{\rm{S}}}+{I}_{{\rm{DFG}}}={c}_{{\rm{S}}}\,{{\mathcal{F}}}_{{\rm{S}}}+{c}_{{\rm{DFG}}}\,{{\mathcal{F}}}_{{\rm{DFG}}}$, respectively, depend on molecule and gold properties at the interfaces of the NPoM cavity. These properties are described by the coefficients *c*_ aS_, *c*_ SFG_, *c*_ S_ and *c*_ DFG_, which are challenging to evaluate. This challenge includes quantifying the non-resonant SFG signal from the gold at the interfaces^25,26^, which adds coherently^27^ to the resonant vibrational SFG signal from the molecular layer introduced in Fig. 1. In this study, we thus discuss and calculate only intensity enhancement factors, as they fully capture the influence of the tip on both the linear and nonlinear optical scattering processes.

**Fig. 2:**
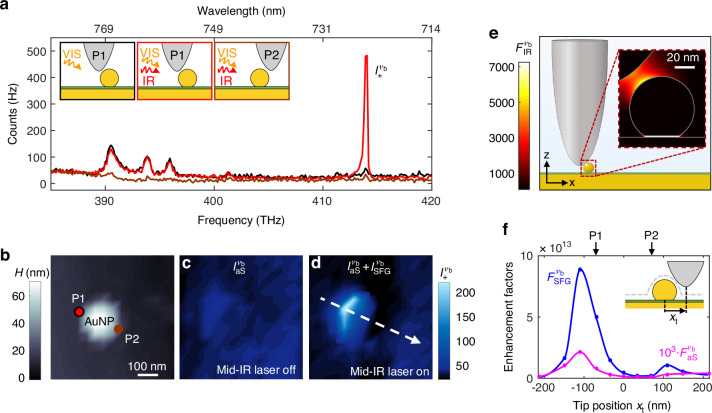
Tip-enhanced nanocavity SFG. **a** Anti-Stokes side of the spectra of a BPT-filled NPoM cavity below an oscillating metallized scanning probe tip. The NPoM is formed by a 63 nm high faceted Au particle on top of an Au mirror that is functionalized with a monolayer of BPT molecules. Insets illustrate the positions P1 and P2 of the tip relative to particle, as well as illumination at VIS (orange) and IR (red) frequencies. Black spectrum is recorded for tip positioned at P1 under VIS illumination exclusively. Red and brown spectra were recorded under VIS and IR (2.2 mW tuned to the vibrational mode *ν*_b_ at 32 THz) illumination at positions P1 and P2, respectively. **b** Topography image of the NPoM cavity. Red and brown dots indicate the tip positions P1 and P2 where spectra shown in panel (**a**) were recorded. Maps of ${I}_{+}^{\,{\nu }_{{\rm{b}}}}$ (averaged over peak area) without (**c**) and with (**d**) IR illumination. VIS illumination is at 382 THz (785 nm) at a power of 200 *μ*W. Acquisition time per spectra is 2 s. Tapping amplitude (TA) is 50 nm. **e** Simulated spatial distribution of the intensity enhancement factor at 32 THz ${F}_{{\rm{IR}}}^{{\nu }_{{\rm{b}}}}$ around the NPoM cavity. **f** Simulated $\mathcal{F}_{\rm{aS}}^{\nu_{\rm{b}}}(\vec{\rm{r}}_{\rm{hs}})$ (pink line) and $\mathcal{F}_{\rm{SFG}}^{\nu_{\rm{b}}}(\vec{\rm{r}}_{\rm{hs}})$ (blue line) enhancement factors along the dashed arrow depicted in (**d**). *x*_t_ is the lateral position of the tip with respect to the particle’s center. The vertical distance Δ*z*_t_ = 20 nm between tip apex and NPoM is kept constant, as illustrated in the inset. Optical maps for other vibrational signals can be found in Supplementary Note 3

In Fig. 2a, we experimentally demonstrate that (i) the tip enables detection of otherwise imperceptible SFG signals from a molecule-filled NPoM cavity under VIS and IR continuous wave illumination and (ii) this signal strongly depends on the mechanical nanopositionning of the tip. To that end, we fabricate NPoMs by functionalizing a template-stripped gold film with a monolayer of biphenyl-4-thiol (BPT) molecules and drop-casting faceted gold particles. Thus, the NPoM gap is fully filled with molecules and the gap height is determined by the monolayer thickness^28^ (see Methods). We probe the NPoM cavities with an s-SNOM, utilizing an oscillating metallized atomic force microscopy (AFM) tip (nano-FTIR tip from attocube systems with nominal apex diameter of 100 nm). In s-SNOM, an off-axis parabolic mirror focuses a laser beam from the side onto the tip and collects the elastically back-scattered light, enabling operation with multiple collinear beams across a wide THz to VIS frequency range. With a grating spectrometer, the setup also detects inelastically scattered light, including Raman signals^29^, making it ideal for our studies. More details about the setup and alignment can be found in the Methods and Supplementary Note 1.

We first place the tip above the NPoM at position P1 (illustrated in the inset of Fig. 2a and indicated in the topography image of Fig. 2b), illuminate the tip above the NPoM cavity with a focused CW VIS laser beam of 382 THz (785 nm) and record a spectrum of the inelastically scattered light in the absence of IR illumination (black spectrum, in Fig. 2a). This spectrum shows the anti-Stokes signals corresponding to different vibrational modes of BPT molecules inside the NPoM cavity. Interestingly, when a CW monochromatic IR illumination is tuned to the molecule’s bending mode, labeled *ν*_b_, at ${\omega }_{{\rm{IR}}}^{{\nu }_{{\rm{b}}}}/(2\pi )=32$ THz (1080 cm^−1^), we observe a remarkable and frequency-selective increase of the intensity on the sideband ${\omega }_{+}^{{\nu }_{{\rm{b}}}}$ (${I}_{+}^{{\nu }_{{\rm{b}}}}\equiv {I}_{+}({\nu }_{{\rm{b}}})$, red spectrum). In agreement with a previous far-field spectroscopy study^13^, the increased peak intensity may result from an enhancement of the resonant SFG signal associated with the vibrational mode *ν*_b_ or, in other words, to a coherent upconversion of IR photons from 32 THz–414 THz mediated by the molecular vibration *ν*_b_. In the future, frequency-scan SFG experiments could be performed to differentiate the vibrational SFG contribution from that of the gold. We note that the peak intensities of the other vibrational sidebands, which are not driven by the CW monochromatic IR illumination, remain unchanged. This observation rules out the possibility that ${I}_{+}^{{\nu }_{{\rm{b}}}}$ is enhanced due to heating effects caused by the IR illumination.

To explore the impact of the tip position on the SFG signals, we record spectra while scanning the tip across the NPoM cavity. At position P2 (brown curve in Fig. 2a), all peaks show a reduced intensity compared to position P1. Mapping the anti-Stokes and SFG signals as function of tip position (Fig. 2c, d) reveals strong SFG signals around P1, whereas anti-Stokes signals remain weak regardless of the tip position. These measurements highlight the dominance of tip-enhanced SFG over anti-Stokes signals and evidence an asymmetry of tip-enhanced SFG with respect to the particle center. This asymmetry is a robust feature observed for many particles we imaged. Few examples are shown in Supplementary Note 4.

The asymmetry of the SFG map of Fig. 2d can be verified by numerical calculations of the signal enhancement factors ${{\mathcal{F}}}_{{\rm{SFG}}}^{{\nu }_{{\rm{b}}}}$ and ${{\mathcal{F}}}_{{\rm{aS}}}^{{\nu }_{{\rm{b}}}}$ as a function of the lateral position *x*_t_ of the tip with respect to the particle’s center. To that end, the tip is modeled as a 1 *μ*m long Pt half ellipsoid with an apex diameter of 100 nm and the NPoM as a truncated 60 nm height Au sphere at a distance of 1.15 nm above an Au film (see schematic of Fig. 2e and Methods). For a given tip position, we calculate the spatial distributions ${F}_{{\rm{IR}}}^{{\nu }_{{\rm{b}}}}(\vec{r})$, ${F}_{{\rm{VIS}}}(\vec{r})$ and ${F}_{+}^{{\nu }_{{\rm{b}}}}(\vec{r})$ around the tip and NPoM cavity. In the inset of Fig. 2e we show, as an example, ${F}_{{\rm{IR}}}^{{\nu }_{{\rm{b}}}}(\vec{r})$ for the tip at position P1. Multiplying the intensity distributions, we extract the maximal enhancement factors for the SFG and aS signals inside the gap of the NPoM cavity (according to Eqs. (1) and (2), see also Supplementary Note 5 and 6). We refer to the position at which the factors are maximal as hot-spots (${\vec{{\rm{r}}}}_{{\rm{hs}}}$) and write the corresponding signal enhancement factors as ${{\mathcal{F}}}_{{\rm{SFG}}}^{{\nu }_{{\rm{b}}}}({\vec{{\rm{r}}}}_{{\rm{hs}}})$ and ${{\mathcal{F}}}_{{\rm{aS}}}^{{\nu }_{{\rm{b}}}}({\vec{{\rm{r}}}}_{{\rm{hs}}})$. The blue curve in Fig. 2f shows ${{\mathcal{F}}}_{{\rm{SFG}}}^{{\nu }_{{\rm{b}}}}({\vec{{\rm{r}}}}_{{\rm{hs}}})$ when the tip is scanned across the particle, as illustrated in the inset. We find that ${{\mathcal{F}}}_{{\rm{SFG}}}^{{\nu }_{{\rm{b}}}}({\vec{{\rm{r}}}}_{{\rm{hs}}})$ is asymmetric with respect to the particle, confirming the experimental SFG results (Fig. 2d). Further, we find that the SFG signal enhancement can reach up to 14 orders of magnitude for a small range of tip positions close to P1. For comparison, the pink curve in Fig. 2f depicts the simulated linescan of the anti-Stokes signal enhancement, ${{\mathcal{F}}}_{{\rm{aS}}}^{{\nu }_{{\rm{b}}}}({\vec{{\rm{r}}}}_{{\rm{hs}}})$ at the frequency of the vibrational mode *ν*_b_. ${{\mathcal{F}}}_{{\rm{SFG}}}^{{\nu }_{{\rm{b}}}}({\vec{{\rm{r}}}}_{{\rm{hs}}})$ is increased by 3–4 orders of magnitude compared to ${{\mathcal{F}}}_{{\rm{aS}}}^{{\nu }_{{\rm{b}}}}({\vec{{\rm{r}}}}_{{\rm{hs}}})$, owing to the infrared intensity enhancement provided by the tip-enhanced NPoM cavity. Interestingly, ${{\mathcal{F}}}_{{\rm{aS}}}^{{\nu }_{{\rm{b}}}}({\vec{{\rm{r}}}}_{{\rm{hs}}})$ qualitatively follows the ${{\mathcal{F}}}_{{\rm{SFG}}}^{{\nu }_{{\rm{b}}}}({\vec{{\rm{r}}}}_{{\rm{hs}}})$ linescan, that is, it exhibits a maximum near tip position P1 and a similar asymmetry with respect to the particle (alike results of Fig. 2c). This maximum is 17 times higher than the anti-Stokes signal enhancement of the NPoM cavity alone (see Fig. 4), indicating that the tip also enhances near fields in the NPoM gap at visible frequencies. We note that the asymmetry of both SFG and aS linescans can be attributed to the combined and enhanced longitudinal and transversal response of the tip under visible illumination (see Supplementary Note 8).

**Fig. 3:**
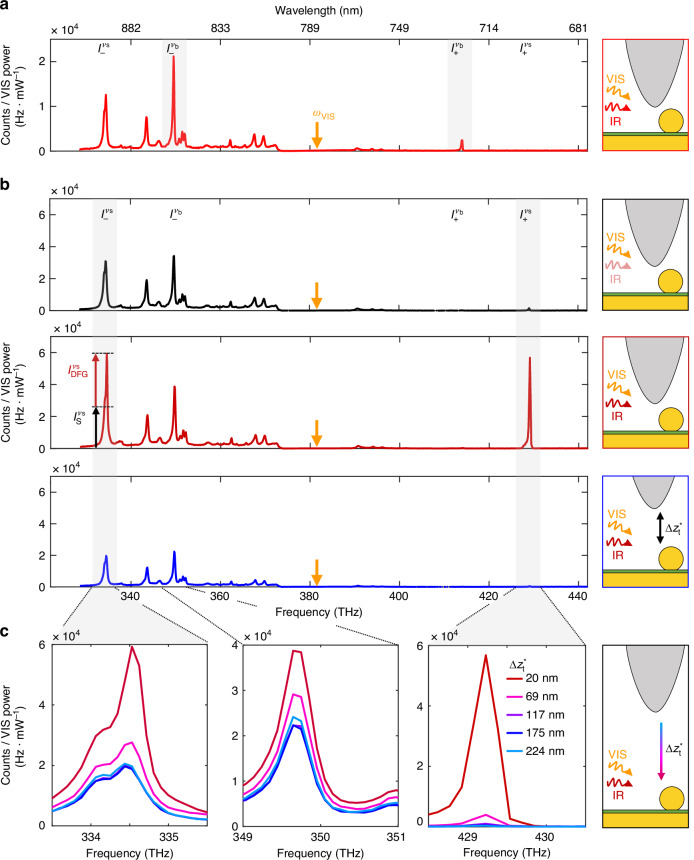
Illumination- and tip-controlled spectra from NPoM cavities. **a** Spectrum of a BPT-filled NPoM cavity below an oscillating metallized scanning probe tip in intermittent contact mode at position P1 under VIS (200 *μ*W at 382 THz / 785 nm, marked *ω*_VIS_) and intense IR (6.2 mW tuned to the vibrational mode *ν*_b_ at 32 THz / 1080 cm^−1^) illumination. Data are the same as the one shown in Fig. 2a. **b** Spectra of BPT molecules in another NPoM cavity below an oscillating metallized scanning probe tip. Black spectrum was recorded for tip in intermittent contact mode ($\Delta {z}_{{\rm{t}}}^{* }=20$ nm) under VIS (250 *μ*W at 382 THz) and weak IR (0.2 mW tuned to the vibrational mode *ν*_s_ at 48 THz / 1585 cm^−1^) illumination. Red and blue spectra were recorded under VIS and intense IR (5.8 mW) illumination for tip in intermittent contact mode ($\Delta {z}_{{\rm{t}}}^{* }=20$ nm) and retracted ($\Delta {z}_{{\rm{t}}}^{* }=175$ nm), respectively. **c** Tip-height variation of signal intensities around specific vibrational sidebands for 5.8 mW IR illumination. From left to right, we show ${I}_{-}^{\,{\nu }_{{\rm{s}}}}$, ${I}_{-}^{\,{\nu }_{{\rm{b}}}}$ and ${I}_{+}^{\,{\nu }_{{\rm{s}}}}$. Sketches illustrate the tip at position P1 for two tip-NPoM effective distances $\Delta {z}_{{\rm{t}}}^{* }$ (in intermittent contact mode and retracted) as well as illumination at VIS (orange) and IR (red) frequencies. Acquisition time for spectra is 2 s. Tapping amplitude (TA) is 50 nm

Tip-enhanced nanocavities can be applied to study nonlinear CW optical signals from nanoscale areas of molecular monolayers across a broad infrared spectral range. This is illustrated in Fig. 3, where two spectra recorded at tip position P1 are compared. In Fig. 3a, we show the spectrum obtained under illumination at ${\omega }_{{\rm{IR}}}^{{\nu }_{{\rm{b}}}}/(2\pi )=32$ THz (same as in Fig. 2a). In Fig. 3b, a different vibrational mode of the BPT molecules is driven by IR illumination from a quantum cascade laser (QCL). For the latter, we selected the molecular stretching mode (labeled *ν*_s_) at ${\omega }_{{\rm{IR}}}^{{\nu }_{{\rm{s}}}}/(2\pi )=48$ THz. The red spectrum in Fig. 3b shows that the intensity at the sideband ${\omega }_{+}^{{\nu }_{{\rm{s}}}}$ is enhanced by a factor of 36 compared to the black spectrum in Fig. 3b when the QCL illumination power is increased by a factor of 32. The two driven sidebands shown in Fig. 3a, b differ by 16 THz (500 cm^−1^), indicating that the tip efficiently enhances IR radiation and thus SFG signals over a broad spectral range. Interestingly, IR illumination at ${\omega }_{{\rm{IR}}}^{{\nu }_{{\rm{s}}}}$ also increases the intensity of the ${\omega }_{-}^{{\nu }_{{\rm{s}}}}$ sideband (Fig. 3b), while illumination at ${\omega }_{{\rm{IR}}}^{{\nu }_{{\rm{b}}}}$ increases the intensity of the ${\omega }_{-}^{{\nu }_{{\rm{b}}}}$ sideband (Fig. 3a). This demonstrates frequency-selective CW tip-enhanced DFG alongside CW tip-enhanced SFG.

Employing a metallic scanning probe tip, we can control in-operando the intensity enhancement factor inside the NPoM gap. For an experimental demonstration, we record spectra while retracting the oscillating tip. Fig. 3c shows close-ups of vibrational signals at the sidebands ${\omega }_{-}^{{\nu }_{{\rm{b}}}}$ (only Stokes signal), ${\omega }_{-}^{{\nu }_{{\rm{s}}}}$ (DFG and Stokes signals) and ${\omega }_{+}^{{\nu }_{{\rm{s}}}}$ (comprising SFG and a marginal anti-Stokes contribution) for various effective vertical tip-NPoM distances ($\Delta {z}_{{\rm{t}}}^{* }$, see Methods). We observe that the SFG signal is efficiently controlled over several orders of magnitude, vanishing to the noise limit when the oscillating tip is retracted by >250 nm. Further, the driven ${I}_{-}^{{\nu }_{{\rm{s}}}}$ and non-driven ${I}_{-}^{\nu_{\rm{b}}}$ signals can be controlled too. Although they decrease with increasing $\Delta {z}_{{\rm{t}}}^{* }$, they do not vanish completely. We attribute the residual signals to those originating from the NPoM cavity in absence of tip and refer to them as background signals *I*_−,bg_. Overall, tip nanopositioning allows for increasing nonlinear optical signals via controlling intensity enhancement factors rather than by augmenting the illumination power. Tip-enhanced NPoM cavities offer therefore a means for efficient IR to VIS coherent upconversion, not only preventing heating of delicate NPoM cavities^28^, but also offering unprecedented opportunities for active tuning of the coupling regime^30,31^ between nanocavity and molecular vibration^32^.

**Fig. 4:**
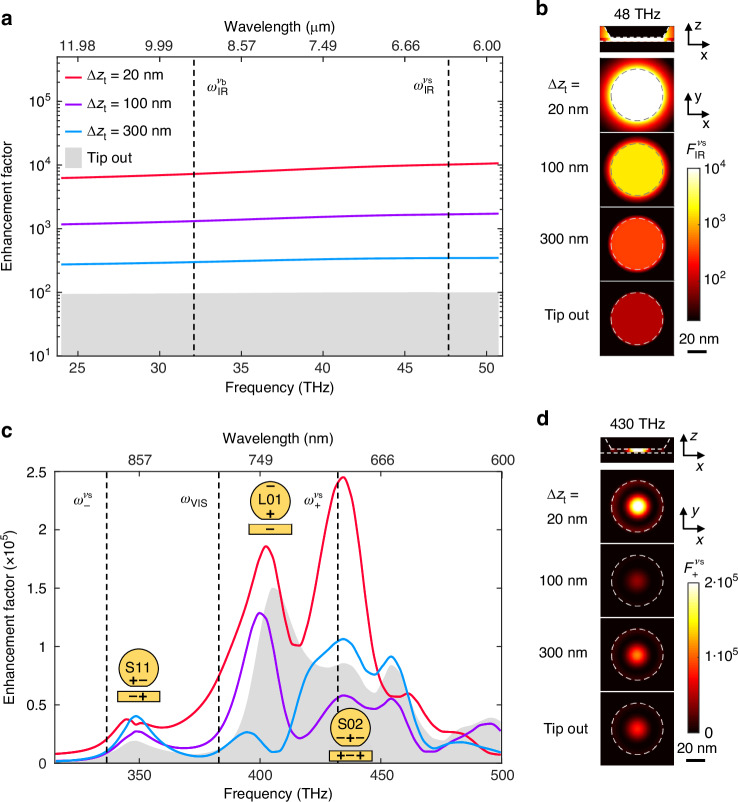
Calculated multispectral enhancement factors inside NPoM cavities. Spectra of the simulated maximum intensity enhancement factor inside the NPoM cavity with a static Pt tip at position P1 in the infrared (**a**) and visible (**c**) frequency ranges for different tip-NPoM distances. Spectra of the NPoM cavity in absence of a tip are depicted by a gray area. Charge distributions of the NPoM cavity modes *S*_11_, *L*_01_ and *S*_02_ are sketched near their resonance frequencies. Panels (**b**, **d**) depict the spatial distribution of the intensity enhancement factor at different frequencies: ${\omega }_{{\rm{IR}}}^{{\nu }_{{\rm{s}}}}/(2\pi )=48$ THz in (**b**) and ${\omega }_{+}^{{\nu }_{{\rm{s}}}}/(2\pi )=430$ THz in (**d**). Top maps show a vertical cross-section of the distribution of the intensity enhancement factor inside the NPoM gap for a tip at a distance of Δ*z*_t_ = 20 nm from the NPoM cavity. Maps below show the intensity enhancement factor distribution on the mid-gap horizontal plane of the NPoM gap for tip-NPoM distances of Δ*z*_t_ = {20, 100, 300} nm and for the NPoM cavity without tip case

To verify and better understand the influence of the tip on both the linear and nonlinear optical signals, we calculate (see Fig. 2e) the intensity enhancement distributions inside the NPoM gap, $F(\vec{{\rm{r}}})$, for various tip-NPoM distances Δ*z*_t_ at IR (Fig. 4b) and VIS (Fig. 4d) frequencies. Plotting the maxima of intensity enhancement factors, $F({\vec{{\rm{r}}}}_{{\rm{hs}}})$, as a function of frequency (Fig. 4a), we observe an increase of about two orders of magnitude across the whole mid-IR spectral range when the tip approaches the NPoM cavity, which we attribute to the lightning rod effect. The highly efficient IR near-field illumination of the NPoM cavity thus explains the remarkable boost of the SFG and DFG signals at the frequencies of the molecular vibrational modes *ν*_b_ and *ν*_s_. In the visible spectral range, the spectral behavior of $F({\vec{{\rm{r}}}}_{{\rm{hs}}})$ is much richer, owing to the presence of the different plasmonic modes supported by the NPoM cavity (gray area in Fig. 4c). In addition to the longitudinal dipolar antenna mode (see inset, labeled *L*_01_), the faceted nanoparticle supports transversal metal-insulator-metal gap modes of different orders and azimuthal symmetry (see inset, labeled *S*_*mn*_)^33,34^. When the tip is approached towards the NPoM cavity, we observe a frequency-dependent non-monotonic behavior of $F({\vec{{\rm{r}}}}_{{\rm{hs}}})$ for intermediate distances 40 nm ≲ *Δz*_t_ ≲ 300 nm, which we attribute to the interference between the tip’s near fields and the far-field illumination at the position of the NPoM cavity (see Supplementary Note 10). For that reason, vibrational signals may not increase monotonically with decreasing Δ*z*_t_, as for example observed in Fig. 3c for the non-driven Stokes signal ${I}_{-}^{{\nu }_{{\rm{b}}}}$. More importantly, for distances Δ*z*_t_ ≲ 40 nm, we find a strong increase of $F({\vec{{\rm{r}}}}_{{\rm{hs}}})$ for most frequencies. This explains why all Stokes and anti-Stokes signals observed in Fig. 3 are larger as the tip approaches, independent of whether they are driven by IR fields or not.

**Fig. 5:**
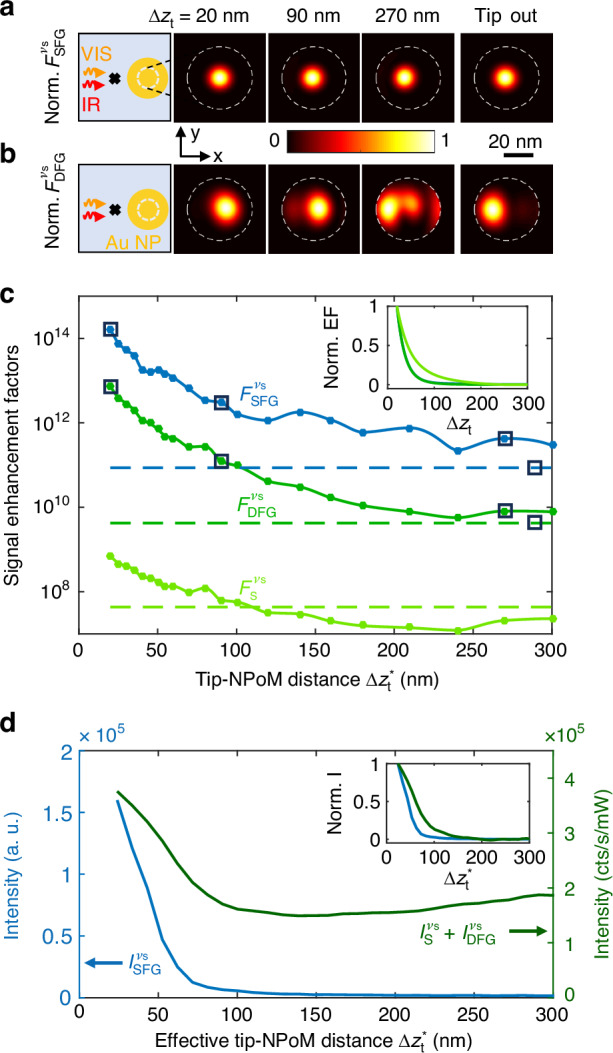
Scaling of tip-enhanced optical signals with tip-NPoM distance. Numerical calculations of normalized SFG (**a**) and DFG (**b**) enhancement factor distributions on the mid-gap horizontal plane of the NPoM gap for different tip-NPoM distances Δ*z*_t_. Bottom facet of the gold nanoparticle is depicted by a white dashed line. First column displays a top-view schematics of the tip-NPoM configuration and the illumination considered. **c** Simulated tip-height dependence of SFG (${{\mathcal{F}}}_{{\rm{SFG}}}^{{\nu }_{{\rm{s}}}}({\vec{{\rm{r}}}}_{{\rm{hs}}})$, blue curve), DFG (${{\mathcal{F}}}_{{\rm{DFG}}}^{{\nu }_{{\rm{s}}}}({\vec{{\rm{r}}}}_{{\rm{hs}}})$, green) and Stokes (${{\mathcal{F}}}_{{\rm{S}}}^{{\nu }_{{\rm{s}}}}({\vec{{\rm{r}}}}_{{\rm{hs}}})$, light green) enhancement factors at the frequency of the vibrational mode *ν*_s_. Inset illustrates the three normalized enhancement factors after smoothing the numerical results with a Whittaker algorithm. **d** Sidebands signals (area under the peaks) extracted from the spectra shown in Fig. 3(b) as a function of effective tip-NPoM distance $\Delta {z}_{{\rm{t}}}^{* }$. Signal intensity measured on the driven *ν*_s_ upper sideband is shown in blue (${I}_{{\rm{SFG}}}^{\,{\nu }_{{\rm{s}}}}$). The intensity measured on the *ν*_s_ lower sideband is shown in dark green (${I}_{{\rm{S}}}^{\,{\nu }_{{\rm{s}}}}+{I}_{{\rm{DFG}}}^{\,{\nu }_{{\rm{s}}}}$). Inset illustrates the normalized behavior of the measured signals after linear background suppression

From the intensity enhancement factor distributions inside the NPoM gap, we calculate the spatial distributions of ${{\mathcal{F}}}_{{\rm{SFG}}}^{{\nu }_{{\rm{s}}}}(\vec{r})$, ${{\mathcal{F}}}_{{\rm{DFG}}}^{{\nu }_{{\rm{s}}}}(\vec{r})$ (Fig. 5a, b) and ${{\mathcal{F}}}_{{\rm{S}}}^{{\nu }_{{\rm{s}}}}(\vec{r})$ to determine ${{\mathcal{F}}}_{{\rm{SFG}}}^{{\nu }_{{\rm{s}}}}({\vec{{\rm{r}}}}_{{\rm{hs}}})$, ${{\mathcal{F}}}_{{\rm{DFG}}}^{{\nu }_{{\rm{s}}}}({\vec{{\rm{r}}}}_{{\rm{hs}}})$ and ${{\mathcal{F}}}_{{\rm{S}}}^{{\nu }_{{\rm{s}}}}({\vec{{\rm{r}}}}_{{\rm{hs}}})$ as a function of tip-NPoM distance Δ*z*_t_. ${{\mathcal{F}}}_{{\rm{SFG}}}^{{\nu }_{{\rm{s}}}}({\vec{{\rm{r}}}}_{{\rm{hs}}})$ increases by about two orders of magnitude for decreasing Δ*z*_t_ (blue curve in Fig. 5c), reaching a remarkable value of about 10^14^ for Δ*z*_t_ = 20 nm, whereas the spatial distribution ${{\mathcal{F}}}_{{\rm{SFG}}}^{{\nu }_{{\rm{s}}}}(\vec{{\rm{r}}})$ remains unchanged as compared to the NPoM without tip (Fig. 5a). A similarly strong increase is observed for ${{\mathcal{F}}}_{{\rm{DFG}}}^{{\nu }_{{\rm{s}}}}({\vec{{\rm{r}}}}_{{\rm{hs}}})$ (green curve in Fig. 5c), reaching a value about 10^13^ for small Δ*z*_t_. However, the spatial distribution of ${{\mathcal{F}}}_{{\rm{SFG}}}^{{\nu }_{{\rm{s}}}}(\vec{{\rm{r}}})$ changes with decreasing Δ*z*_t_ (Fig. 5b), showing that the tip can not only be used for enhancing signals, but also for controlling the position of hot spots within the NPoM gap. In the future, and in case of heterogeneously filled NPoM cavities, such hot-spot control could be used for in-operando selection of specific areas, or even single molecules, to which the cavity couples to.

We highlight that the decrease of SFG and DFG signal enhancement factors with increasing tip-NPoM distance Δ*z*_t_ is equal, but steeper than the Stokes signal enhancement factor (see normalized curves in inset of Fig. 5c). This key advantage of nonlinear signals (e.g. for better spatial localization of an optical response^35–37^) is demonstrated experimentally in Fig. 5d, where we plot the measured intensities ${I}_{+}^{{\nu }_{{\rm{s}}}}={I}_{{\rm{SFG}}}^{{\nu }_{{\rm{s}}}}+{I}_{{\rm{aS}}}^{{\nu }_{{\rm{s}}}} \sim {I}_{{\rm{SFG}}}^{{\nu }_{{\rm{s}}}}$ and ${I}_{-}^{{\nu }_{{\rm{s}}}}={I}_{{\rm{DFG}}}^{{\nu }_{{\rm{s}}}}+{I}_{{\rm{S}}}^{{\nu }_{{\rm{s}}}}+{I}_{{\rm{-,bg}}}^{{\nu }_{{\rm{s}}}}$ as a function of the effective tip-NPoM distance $\Delta {z}_{{\rm{t}}}^{* }$ (see Methods). After subtraction of the background signal from the NPoM cavity, ${I}_{{\rm{-,bg}}}^{{\nu }_{{\rm{s}}}}$, and normalization of ${I}_{+}^{{\nu }_{{\rm{s}}}}$ and ${I}_{-}^{{\nu }_{{\rm{s}}}}$ (inset of Fig. 5d), we clearly see that ${I}_{+}^{{\nu }_{{\rm{s}}}}$ decays much stronger with increasing $\Delta {z}_{{\rm{t}}}^{* }$ than ${I}_{-}^{{\nu }_{{\rm{s}}}}$, demonstrating a different scaling of both signals: ${I}_{+}^{{\nu }_{{\rm{s}}}}$ is a purely nonlinear SFG signal, whereas ${I}_{-}^{{\nu }_{{\rm{s}}}}$ comprises both a nonlinear DFG and linear Stokes signals. We also highlight the increase of ${I}_{{\rm{SFG}}}^{{\nu }_{{\rm{s}}}}$ by about two orders of magnitude when the tip is approached to the NPoM cavity (blue curve in Fig. 5d), being in good agreement with the calculated SFG signal enhancement factor ${F}_{{\rm{SFG}}}^{{\nu }_{{\rm{s}}}}({\vec{{\rm{r}}}}_{{\rm{hs}}})$ (blue curve in Fig. 5c).

Figure 5 demonstrates that tip-enhanced nonlinear SFG signals offer distinct advantages compared to tip-enhanced linear Raman signals. We find a significantly stronger signal enhancement factor, paired with a more pronounced decay as the tip-sample distance increases, which requires a closer proximity of the tip to the object (in our study the NPoM cavity) for signal acquisition. Further, we do not observe SFG background signals generated by the far-field illumination. We thus envision tip-enhanced continuous wave SFG spectroscopy for background-free nanoimaging of molecules or 2D materials on bare substrates. This could be achieved with scanning probe tips providing sufficiently large field enhancement at their apex at both visible and infrared frequencies, eliminating the need for NPoM cavities.

## Discussion

In summary, we have introduced in-operando control of SFG from molecule-filled NPoM cavities via the tip of a scattering-type scanning near-field optical microscope (s-SNOM), adding a versatile technique to the toolbox of nonlinear nanooptics^38–41^. Remarkably, low-power continuous wave illumination at both visible and mid-infrared frequencies is sufficient to access second-order nonlinearities of individual molecule-filled nanocavities, which in future could be applied for studying molecular self-assembled monolayers domains^42^ or even for characterizing the anharmonicities of molecular vibrations and their intramolecular vibrational relaxation mechanisms^43,44^. Our approach thus circumvents various typical challenges of nonlinear nanooptics, such as establishing precise temporal overlap of short high-power laser pulses. Further, our prototypical realization of a cascaded nanolens^18^ allows for deep sub-wavelength field concentration across the whole visible to terahertz spectral range, greatly relaxing the requirements on samples that can be studied via nonlinear spectroscopy. In combination with extreme near-field concentration provided by atomic scale protrusions inside the NPoM gap^45^, SFG involving dipole-forbidden contributions may become a promising topic of research. In the future, optimized scanning probe tips exhibiting simultaneously strong resonances at both IR and visible frequencies may pave the way to v-SFG spectroscopy without the need of NPoM cavities and thus for v-SFG nanoimaging.

## Materials and Methods

### Setup description

A schematic of the setup and a detailed description of its elements is provided in Supplementary Note 1. We use a commercial s-SNOM (neascope, attocube systems) is based on an AFM, whose metallic tip is used to enhance the incident VIS and IR fields at its apex (nano-FTIR tips with nominal tip apex diameter of 100 nm). In proximity to an NPoM cavity, the tip’s near field provides a strongly concentrated illumination in addition to the incident far-field illumination. In our experiment, the sample and tip are illuminated from the side with radiations from CW monochromatic IR and VIS laser sources. The visible beam is first spectrally filtered with a laser line bandpath filter in order to allow for Raman and photoluminescence (PL) spectroscopy measurements. Both collimated beams pass then through beam expanders (reaching 1 cm beam diameter) and attenuators before being combined on a dichroic plate and sent to the high-NA off-axis parabolic mirror (PM) or our s-SNOM. The PM tightly focuses the laser beams onto the oscillating AFM tip (frequency Ω ~ 250 kHz, tapping amplitude TA ~ 50 nm) and collects the backscattered light. To precisely focus both beams onto the tip apex, we record the IR and VIS elastically scattered fields using a MCT detector and a photomultiplier tube (PMT), respectively, in the same fashion as in self-homodyne s-SNOM operation. That is, we optimize the detector signal that is recorded without interferometer and demodulated at the third or fourth harmonic of the tip oscillation frequency, 3 Ω or 4 Ω, respectively. After both VIS and IR s-SNOM signals are maximized, the mirror in front of the PMT is flipped such that the back-scattered visible light is guided to a grating spectrometer for Raman or PL measurements. The VIS excitation and radiation from the AFM deflection laser are removed by placing corresponding filters (NF/LP+SP) in front of the spectrometer entrance.

### Tip-based approach curves (TBAC)

In contrast to standard s-SNOM, the oscillating tip rather than the sample is retracted/approached in our experiments to ensure that changes of the optical signals are not caused by movement of the NPoM cavity within the focus of the far-field illumination. To that end, we apply a linear voltage ramp to the piezo (in the AFM head) onto which the AFM cantilever is mounted. This linear voltage ramp is applied as an offset voltage additional to the sinusoidal voltage for exciting the cantilever (tip) oscillation. For converting the piezo voltage *U*_*t*_ into tip-sample distance Δ*z*_tbac_, we record a TBAC on the Au mirror next to the AuNP and a standard approach curve using the calibrated sample scanner. Comparison of both approach curves (recorded with the same parameters and alignment) allows for converting *U*_*t*_ to Δ*z*_tbac_. The corresponding microscope script is available in the Zenodo repository.

### Effective tip-NPoM distance $\Delta {z}_{{\rm{t}}}^{* }$

In order to compare the experimental results obtained with a tip oscillating at tapping amplitude TA = 50 nm and the numerical calculations performed with a static tip, we introduce the offset distance ${z}_{{\rm{t,0}}}^{* }=20$ nm. The offset is chosen such that numerical calculations for a tip-NPoM distance of ${z}_{{\rm{t,0}}}^{* }$ reproduce faithfully the spatial variations of optical signals *I*_SFG_, *I*_DFG_, *I*_aS_ and *I*_S_ observed experimentally with an oscillating tip in intermittent contact mode. The effective tip-NPoM distance can consequently be defined as: $\Delta {z}_{{\rm{t}}}^{* }=\Delta {z}_{{\rm{tbac}}}+{z}_{{\rm{t,0}}}^{* }$, with Δ*z*_tbac_ the variation of distance recorded during a TBAC (see section above).

### Sample preparation

A detailed description of the different sample preparation steps can be found elsewhere^46^. In short, template-stripped gold films are incubated for 3 h in a biphenyl-4-thiol (BPT) solution (ethanol buffer, Sigma-Aldrich), yielding a self-assembled monolayer (SAM) of BPT. Subsequently, faceted gold nanoparticles (BBI solutions, 80 nm nominal diameter) are drop-casted on the BPT functionalized gold films. After incubation of 10–30 min (depending on the concentration of NP solution), the samples are gently rinsed with DI water and cleaned with nitrogen gas. Far-field optical characterization of the obtained NPoM samples show that the particles strongly enhance the Raman signal of the molecules, demonstrating that they act as nanocavities (Supplementary Note 2).

### Numerical simulations

The electromagnetic simulations are performed with a commercial FEM package (COMSOL). The simulation universe contains a Pt tip, modeled as a 1 *μ*m height half ellipsoid with 100 nm apex diameter, a gold nanoparticle with a facet size of *w* = 35 nm and a height of *h* = 60 nm, which is separated from a 150 nm thick gold film by a *d* = 1.15 nm thick dielectric layer with refractive index *n* = 1.4 (see Supplementary Note 7 of SI for a justification of the choice of the tip height and tip apex geometry). The combination of optical gap size (*n* ⋅ *d*) and particle’s facet size (*w*) are chosen to fit well the AFM and photoluminescence signals of representative NPoM cavities under our microscope scanning tip (cf. Supplementary Note 9 in SI). The simulation universe is surrounded by a 400 nm thick PML layer. Special care is taken to mesh the near-contact regions of our cascaded nanostructure. The dielectric function of gold is obtained by linearly interpolating the data provided in ref. ^47^ for the VIS spectral range and in ref. ^48^ for the IR range. For simplicity, we consider intensity enhancement factors given by $F={(| {E}_{s}| /| {E}_{0}| )}^{2}$ instead of $F={(1+{E}_{s}/{E}_{0})}^{2}$ ^20^ as the scattered fields *E*_*s*_ in the NPoM gap are strongly dominated by the out-of-plane component and much greater than the incident field *E*_*s*_ ≫ *E*_0_. ∣*E*_*s*_∣ is numerically calculated for a plane-wave excitation *E*_0_ incident at an angle of 35 degrees from the surface.

## Supplementary information

Supplementary information for In-operando control of sum-frequency generation in tip-enhanced nanocavities

## Data Availability

The data, plotting scripts, and complementary description of the numerical calculations used in this study are openly available in the Zenodo repository: 10.5281/zenodo.15077282.
