# Supplementary material for: In-operando control of sum-frequency generation in tip-enhanced nanocavities
Source: Light Sci Appl. 2025 May 22;14:203. doi: 10.1038/s41377-025-01855-5 (PMC12098766; doi:10.1038/s41377-025-01855-5)
Supplement: Supplementary file 1 — Supplementary information for In-operando control of sum-frequency generation in tip-enhanced nanocavities [file 41377_2025_1855_MOESM1_ESM.pdf]

Supplementary Information for "In-operando control of sum-frequency generation in tip-enhanced nanocavities"

Philippe Roelli,[1,*] Isabel Pascual Robledo,[1,2] Iris Niehues,[3]
Javier Aizpurua,[4,5,6] and Rainer Hillenbrand[1,5,6,†]

[1] *CIC nanoGUNE BRTA, 20018 Donostia-San Sebastián, Spain*
[2] *Materials Physics Center, CSIC-UPV/EHU, 20018 Donostia-San Sebastián, Spain*
[3] *Institute of Physics, University of Münster, 48149 Münster, Germany*
[4] *Donostia International Physics Center (DIPC), 20018 Donostia-San Sebastián, Spain*
[5] *IKERBASQUE, Basque Foundation for Science, 48013 Bilbao, Spain*
[6] *Department of Electricity and Electronics, University of the Basque Country (UPV/EHU), 48940 Leioa, Spain*

(Dated: March 26, 2025)

* p.roelli@nanogune.eu
† r.hillenbrand@nanogune.eu

Supplementary Note 1. Optical setup

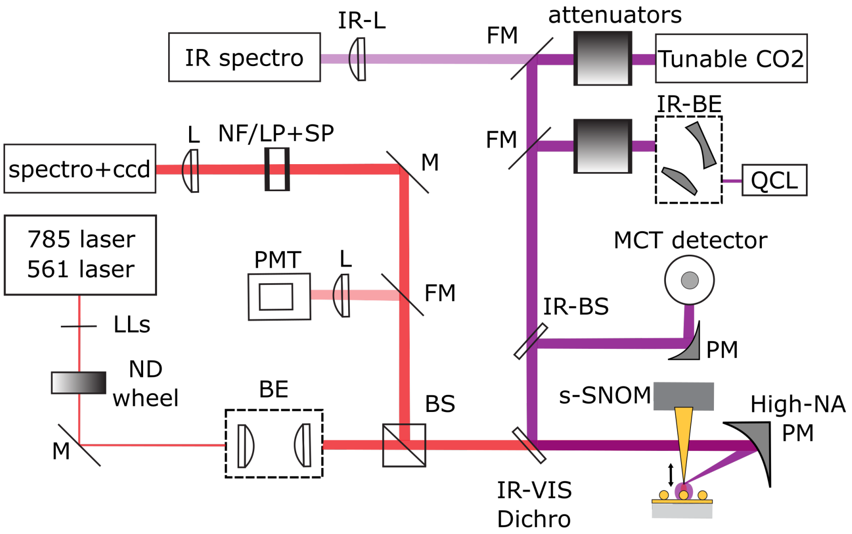

Supplementary Figure S1. **Schematic of the optical setup**. *VIS tip illumination path.* **785 laser**: Raman excitation via 785 nm CW single mode wavelength-stabilized laser diode (SureLock785 from Coherent), **561 laser**: PL excitation via 561 nm CW single wavelength laser diode (Cobolt 08-DPL from Hübner Photonics), **LLs**: laser line bandpass filters for the 785 nm (LL01-785-12.5) and 561 nm lasers (LL02-561-12.5 from Semrock), **ND wheel**: neutral density filter wheel, **BE**: Keplerian type beam expander with additional spatial filtering, **BS**: 90:10 (R:T[%]) non-polarizing broadband cube beamsplitter, **IR-VIS Dichro**: ITO front and AR back coated glass plate used as visible infrared dichroic mirror (from Newport). *IR tip illumination path.* **Tunable CO2**: 9.2-10.8 μm adjustable wavelength CO_2 CW monochromatic gas laser (Merit-G from Access Laser Company), **QCL**: 5.7-6.8 μm tunable external cavity IR CW monochromatic mode-hop-free QCL laser (Tunable Laser System from Daylight Solutions), **IR-BE**: reflective beam expander (BE04 from Thorlabs), **attenuators**: metal grid based variable attenuator for the CO_2 laser path (IR attenuator from Lasnix) and double broadband IR linear polarizers variable attenuator for the QCL path, **IR-BS**: 50:50 (R:T[%]) ZnSe broadband beamsplitter. *Scanning probe setup.* **s-SNOM**: customized scattering-type scanning near-field microscope (NeaSNOM from attocube), being operated with platinum-iridium tips with 100 nm tip apex diameter (nano-FTIR from attocube), **High-NA PM**: silver protected off-axis parabolic mirror (NA$= 0.72$ — NA$_{\text{eff}} \sim 0.55$ for our beam parameters, EFL$\simeq 6.5$ mm). *IR detection path.* **PM**: silver protected parabolic mirror, **MCT detector**: Wideband cooled photovoltaic HgCdTe(MCT) detector with amplifier (KLD-0.1 from Kolmar Technologies), **IR spectro**: Analog CO_2 laser spectrometer (CO_2 Laser Spectrum Analyzer from Optical Engineering). *VIS detection path.* **PMT**: photomultiplier tube (R9110 from Hamamatsu) and variable gain low noise current amplifier (DLPCA-200 from FEMTO Messtechnik), **SP+NF/LP**: shortpass filter for AFM deflection laser suppression (FF01-945/SP-25) and 785 nm notch filter for Raman measurements (NF03-785E-25) or 561 nm longpass filter for PL measurements (BLP02-561R-25 from Semrock), **spectro+ccd**: Visible grating spectrometer with 300 l/mm grating (Kymera 328i from Andor) and CCD camera (DU420A-BEX2-DD from Andor) *Miscellaneous.* **M**: broadband silver mirror, **FM**: flip mirror, **L**: broadband achromatic doublets, **IR-L**: ZnSe broadband plano-convex lens.

Supplementary Note 2. Far-field Raman characterization of the NPoM samples

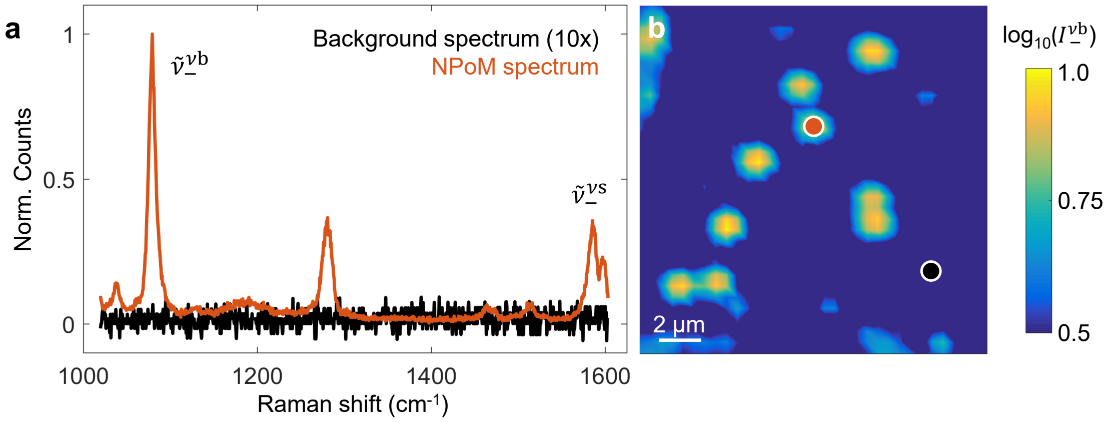

Supplementary Figure S2. **Raman signal of BPT molecules inside NPoM nanocavities**. (a) Surface-enhanced Raman spectrum of different vibrational modes of BPT molecules inside a NPoM nanocavity (normalized, red line). The normalized spectrum (10x amplified) in absence of nanocavity is shown in black for comparison. The spatial location corresponding to the two spectra is depicted on the Raman map of panel (b). (b) The Raman map shows the normalized intensities at the ν_{-}^{b} lower vibrational sideband in logarithmic scale. These far-field measurements are performed with a commercial Renishaw microscope (785 nm illumination at a power of 200 μW, acquisition time per pixel of 0.5 s, objective NA of 0.85). For the measurements shown in the manuscript, isolated nanocavities are selected to avoid contamination of detected signals from other nearby nanocavities.

Supplementary Note 3. Tip-controlled optical signals from a molecule-filled NPoM cavity

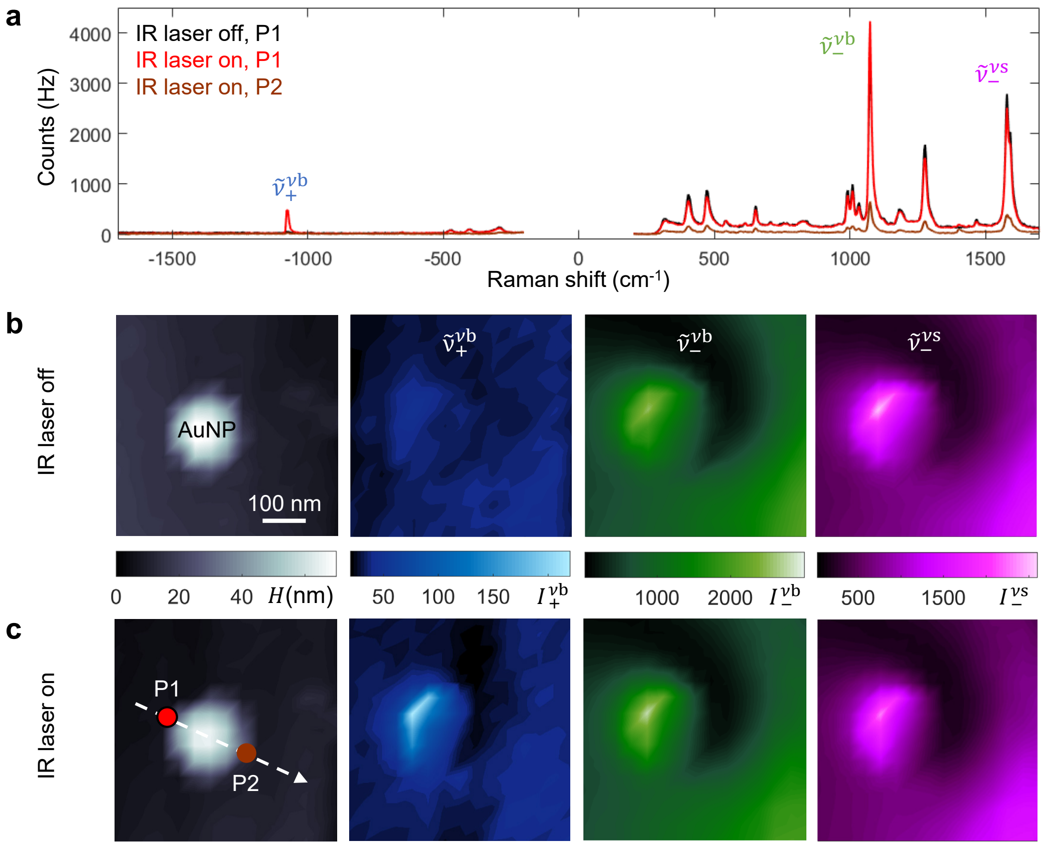

Supplementary Figure S3. **Tip-controlled optical signals**. (a) Spectra of a BPT-filled NPoM cavity (80 nm nominal diameter Au nanosphere) functionalized with BPT molecules below an oscillating scanning tip (100 nm apex diameter). Black spectrum is recorded for tip positioned at position P1 under VIS illumination. Red and brown spectra are recorded under VIS and IR (2.2 mW tuned to the vibrational mode ν_b at 32 THz) illumination at positions P1 and P2, respectively. For a better comparison of the spectra with standard Raman spectra of BPT, we show the spectra in wavenumbers corresponding to the Raman shift. The left part of the red and black spectra are the same data as the ones shown in Fig. 2a. (b,c) Simultaneously recorded topography and optical maps without (b) and with (c) IR illumination. Optical maps at the frequencies $\omega_{+}^{\nu b}/(2\pi) = 414$ THz (corresponding to a Raman shift of -1080 cm^{-1}), $\omega_{-}^{\nu b}/(2\pi) = 350$ THz (1080 cm^{-1}) and $\omega_{-}^{\nu s}/(2\pi) = 335$ THz (1575 cm^{-1}) are shown in blue, green and pink, respectively. Red and brown dots indicate the tip positions P1 and P2 where spectra shown in panel (a) are recorded. The illumination direction is illustrated by the white dashed arrow in (c). VIS illumination is at 382 THz (785 nm) at a power of 200 μW. Acquisition time per spectrum is 2 s. Tapping amplitude (TA) is 50 nm. Topography image and optical maps at frequency $\omega_{-}^{\nu b}/(2\pi)$ are the same as the ones shown in Fig.2c and Fig.2d of the main text.

Supplementary Note 4. Tip-controlled SFG signals from other NPoM cavities

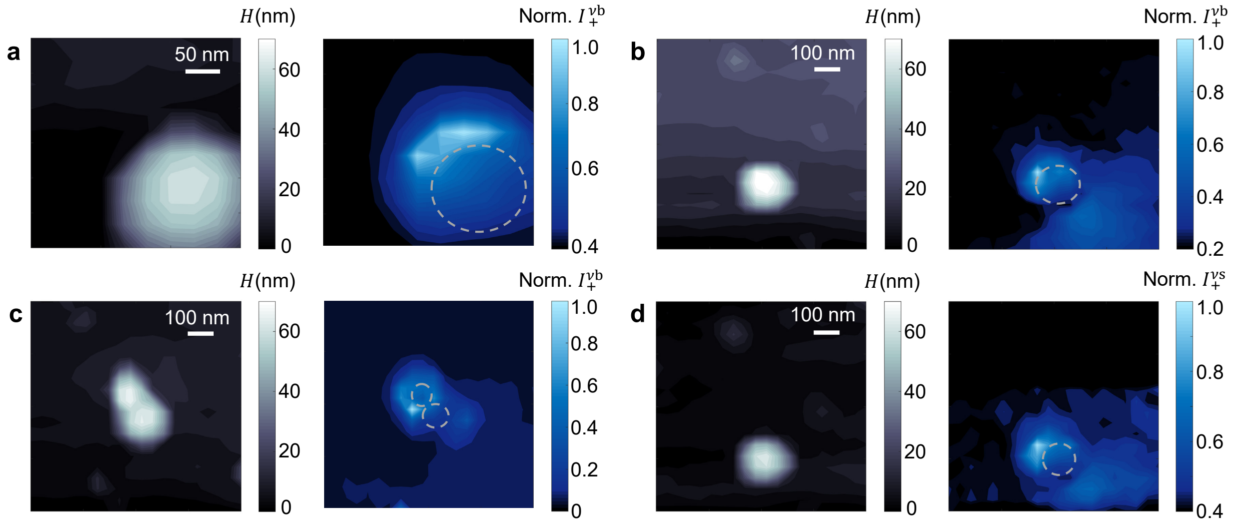

Supplementary Figure S4. **Tip-controlled cavity-enhanced SFG**. Optical maps at IR driven upper vibrational frequencies for other representative NPoM cavities of our samples, under similar illumination direction as in Fig. S3. Each panel is composed in the following way. Right: Topography image of the NPoM cavity. Left: Normalized optical map of the signal I_+ as a function of tip position. The illumination direction is illustrated by the white dashed arrow. For improved clarity, the contour of the nanoparticle is outlined with a dashed gray line on the optical maps. In panels (a-c), the IR illumination is tuned to the vibrational mode ν_b at 32 THz and the optical maps are recorded at $\omega_+^{\nu b}$. In panel (d), the NPoM cavity shown in (b) is illuminated with an IR illumination tuned to the vibrational mode ν_s at 48 THz and the optical map is recorded at $\omega_+^{\nu s}$

.

Supplementary Note 5. Intensity enhancement factors as a function of tip position

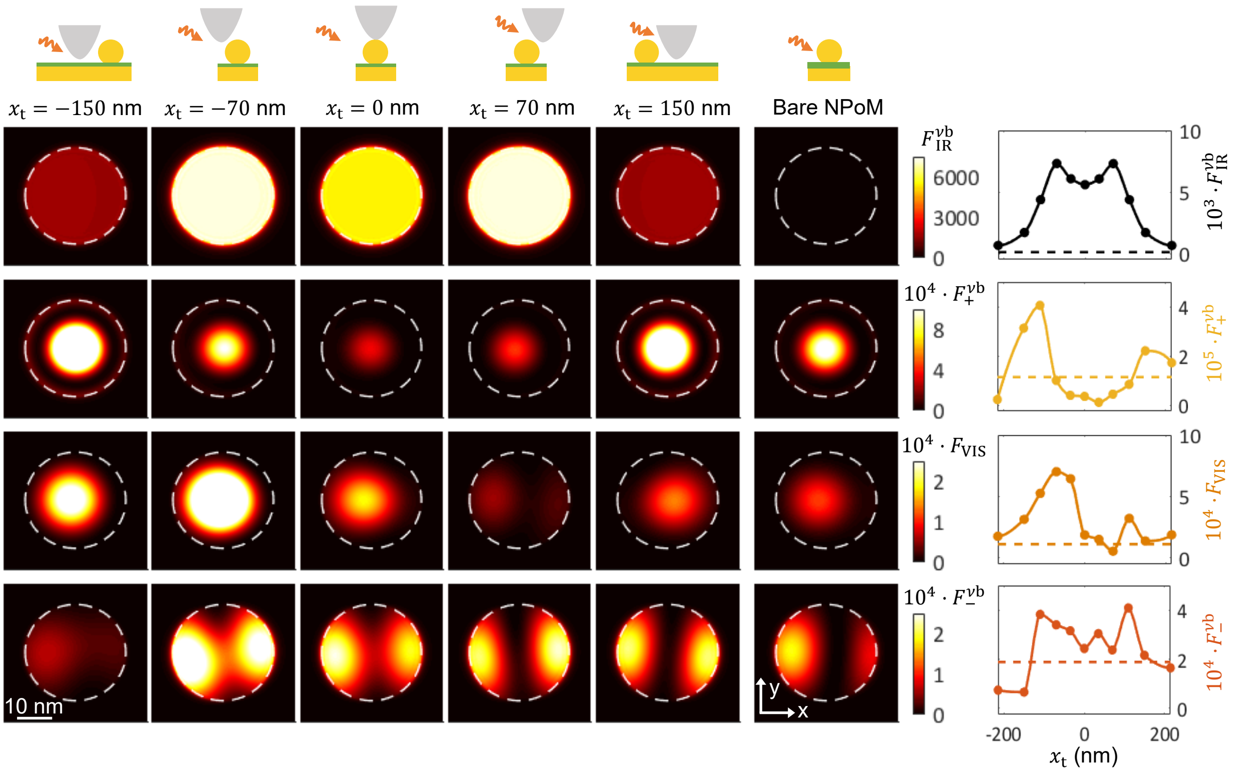

Supplementary Figure S5. **Simulated intensity enhancement factor distributions for different tip positions**. Main panel: Intensity enhancement factor spatial distributions on the mid-gap horizontal plane of the NPoM gap at $\omega_{IR}^{\nu b}$, $\omega_+^{\nu b}$, ω_{VIS} and $\omega_-^{\nu b}$ for tip positions illustrated by the schematics of the top row. $\Delta z_t \simeq 20$ nm for all simulations. The bottom facet of the gold nanoparticle is depicted by a white dashed line. Top row: Sketches of the tip position with respect to the nanoparticle. The orange arrow indicates the illumination direction. Wavelength of VIS illumination is 785 nm. Right column: $F(\vec{r}_{hs})$ as a function of tip position x_t. The dashed horizontal lines depict $F(\vec{r}_{hs})$ for the NPoM cavity without tip.

Supplementary Note 6. Signal enhancement factors as a function of tip position

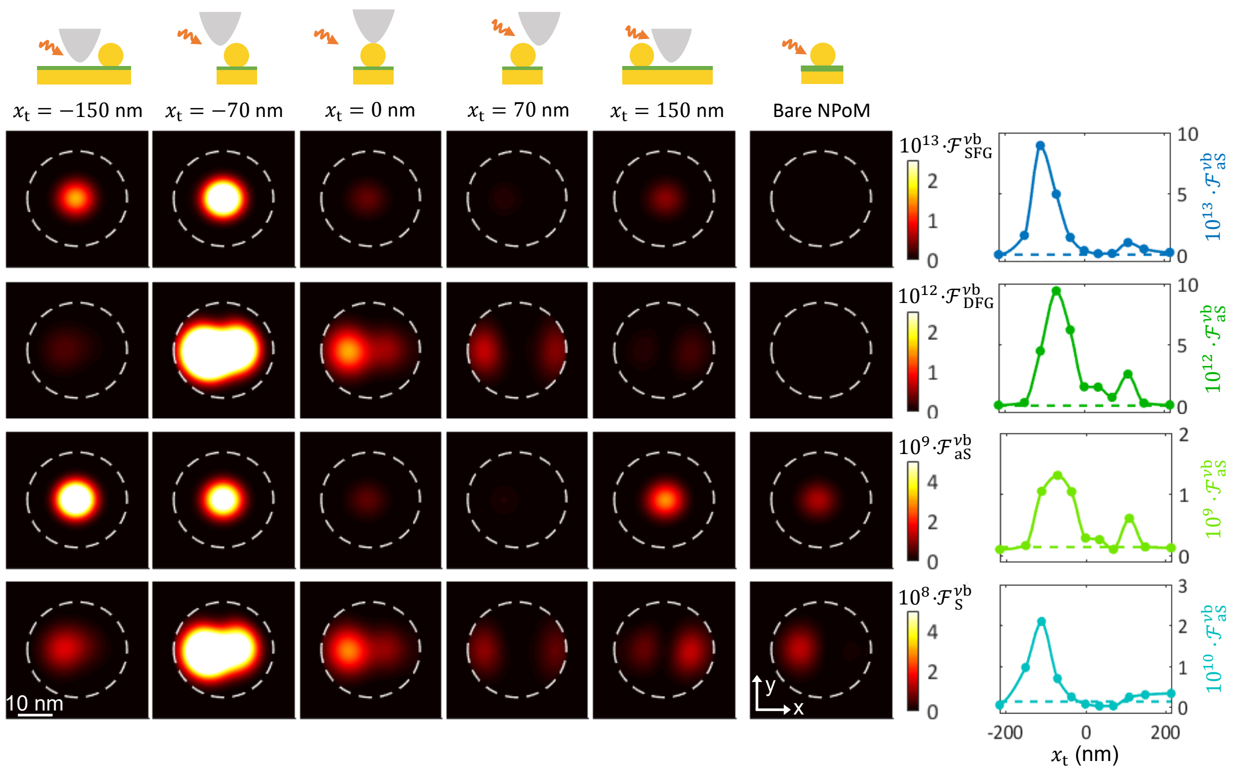

Supplementary Figure S6. **Simulated signal enhancement factor distributions for different tip positions**. Main panel: SFG, DFG, aS and S enhancement factor spatial distributions on the mid-gap horizontal plane of the NPoM gap for tip positions illustrated by the schematics of the top row. $\Delta z_t \simeq 20$ nm for all simulations. The bottom facet of the gold nanoparticle is depicted by a white dashed line. Top row: Sketches of the tip position with respect to the nanoparticle. The orange arrow indicates the illumination direction. Wavelength of VIS illumination is 785 nm. Right column: $\mathcal{F}(\vec{r}_{hs})$ as a function of tip position x_t. The dashed horizontal lines depict $\mathcal{F}(\vec{r}_{hs})$ for the NPoM cavity without tip.

Supplementary Note 7. Modelling of the metal tip

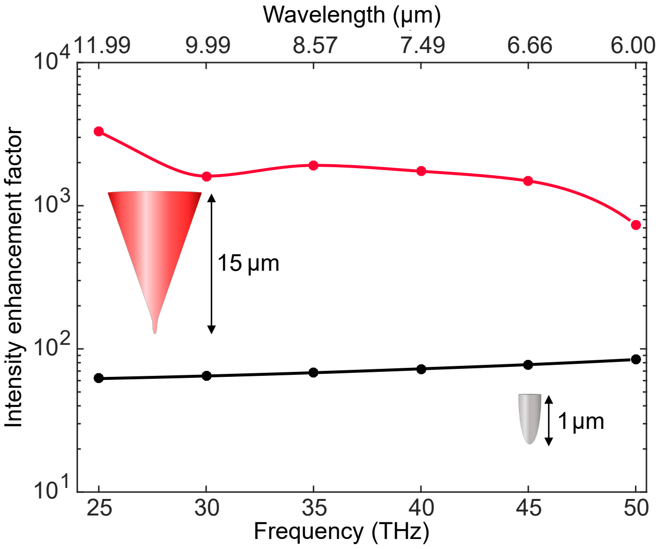

Supplementary Figure S7. **Simulated intensity enhancement factors for long vs short metal tips**. Numerically calculated spectra of the maximum intensity enhancement factor 5 nm below the apex of an isolated tip (i.e. without sample) in the mid-IR frequency range for two tip geometries: the geometry used in the numerical calculations of the manuscript (black line) and a geometry closer to the shape and dimension of the tip used experimentally (red line) [S1]. Sketches illustrate the two different geometries considered. The dimensions of the short tip were chosen so that the heavily multi-scale tip-enhanced NPoM cavity simulations converge. The figure shows that the long tips used in the experiments (15 μm long) may have intensity enhancement factors between 8 and 50 times stronger than the short tips used in simulations. The IR enhancement factors discussed in the main text can therefore be considered as a conservative estimate. The slight spectral variation for the longer tip can be attributed to higher order broad resonances of the long conical tip [S2].

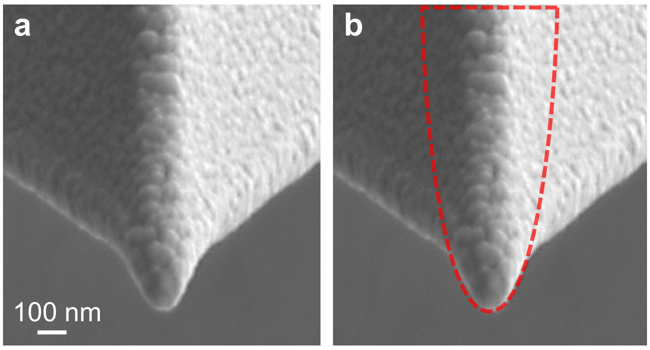

Supplementary Figure S8. **Comparison of tip geometry in experiment and simulation**. (a) Scanning electron microscopy (SEM) image of one nano-FTIR tip used in the experiment captured at a tilt angle of 52°. The image is stretched along the y-axis to compensate for the tilt and to accurately depict the proportions of the tip apex. (b) The geometry of the tip used in the simulations (a 1 μm long half-ellipsoid with 100 nm apex diameter) is depicted with a dashed red line on top of the SEM image presented in (a).

Supplementary Note 8. Spatial distribution of intensity enhancement around the tip

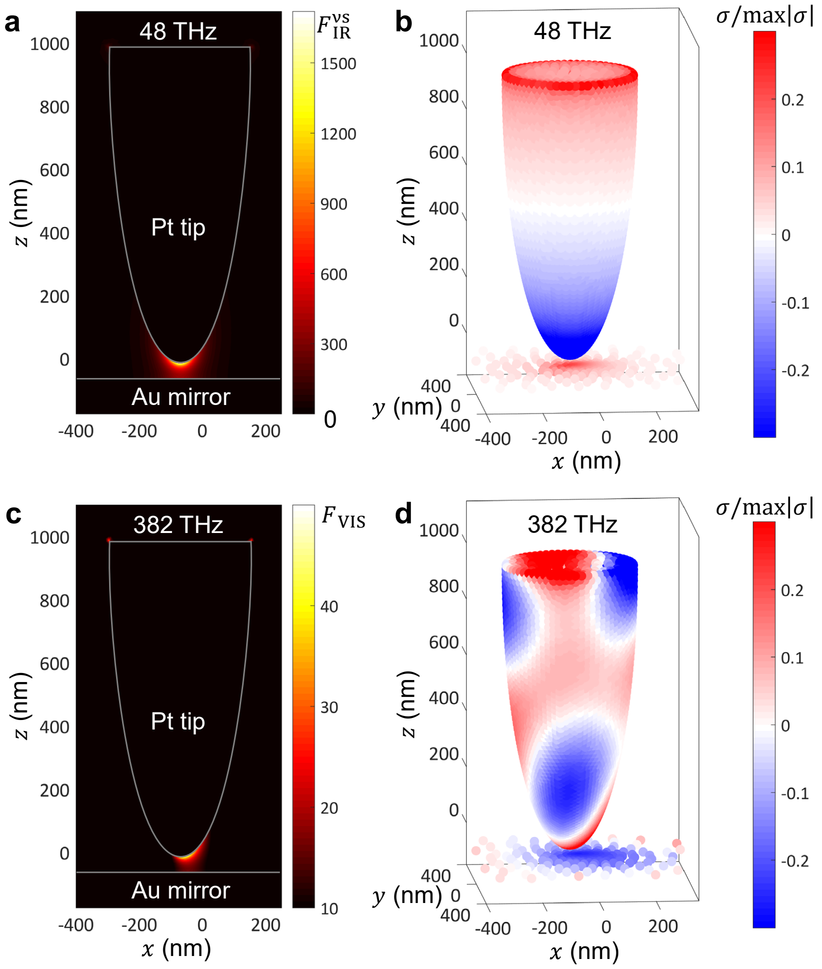

Supplementary Figure S9. **Spatial distribution of intensity enhancement around the tip**. Simulated spatial distribution of the intensity enhancement factors $F_{\rm IR}^{\nu s}$ (a) and $F_{\rm VIS}$ (c) and corresponding surface charges (b,d) around the Pt tip in close proximity to a gold plane ($\Delta z_{\rm t}$ =48 nm) under plane-wave illumination from the left side (35 degrees relative to sample surface) at $\omega_{\rm IR}^{\nu s}$ (a,b) and $\omega_{\rm VIS}$ (c,d). We see that the location of maximum enhancement factor is below the tip apex for the IR calculation but shifted in the direction of illumination for the VIS calculation. This observation can be explained by the surface charge distributions of the tip, which reveal that we have essentially a longitudinal (i.e. along tip axis) mode in IR but both a longitudinal and a transversal mode in VIS. The interference of the two modes leads to constructive interference on the right side of tip apex and to destructive interference on left side of the tip apex. This finding explains why the tip has to be positioned on the left side of the nanoparticle to provide maximum near-field illumination of the NPoM cavity in the VIS and to obtain maximum optical signals, as observed in main text Fig. 2 (c,d) for $I_{+}^{\nu{\rm b}}$ signals.

Supplementary Note 9. Tip-enhanced NPoM photoluminescence

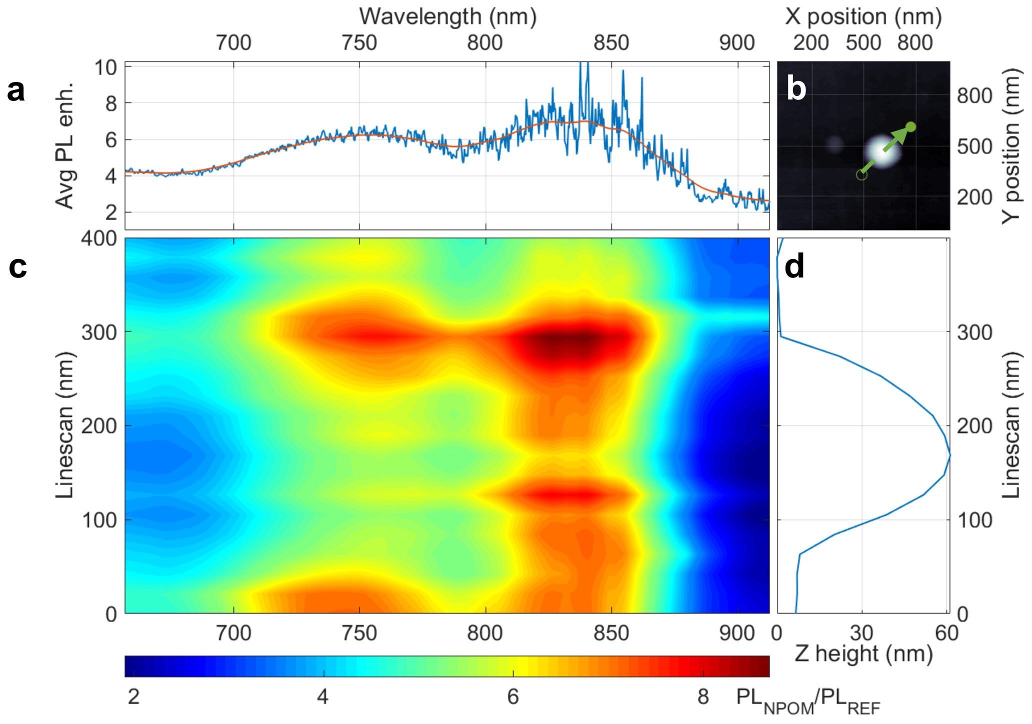

Supplementary Figure S10. **Tip-enhanced NPoM photoluminescence**. In order to evidence the modification of the photonic local density of states (LDOS) of the NPoM cavity modes via the tip, we follow the method presented in Refs. [S3, S4]. Under 561 nm laser illumination, interband transitions occur both in the NPoM cavity and in the bare gold film. The different NPoM modes enhance the gold photoluminescence of the gold film (PL_{REF}), i.e., the radiative recombination of the generated electron-hole pairs. and can be evidenced and characterized by the PL enhancement factor (PL_{NPOM}/PL_{REF}), as shown in this figure and in Fig. S11. Fig. S10 shows the spectral variations of PL_{NPoM}/PL_{REF} under illumination at 561 nm (50 μW) as a function of tip position for an isolated 80 nm gold nanosphere on a BPT functionalized gold film. (a) Averaged PL enhancement over the entire linescan (raw signals in blue, PL results smoothed by a Whittaker filter in red). (b) Topography image of the NPoM. The green arrow depicts the linescan trajectory. (c) Waterfall plot of the PL enhancement as a function of tip position. (d) Height profile acquired from the line scan. Acquisition time per pixel is 50 s, TA = 50 nm. We attribute the two main features of the spectra around 750 nm and 840 nm to the L01 and S11 modes of the NPoM cavity, respectively [S5]. Using these peak positions as well as the height of the NPoM as measured with the AFM (Fig. S10d) in the numerical calculations of the NPoM geometry, we can conclude that the optical gap size ($n \cdot d$) is about 1.61 nm and the particle's facet size (w) is about 35 nm. We observe that the PL signals depend on the tip position, providing an additional evidence of the influence of the tip on the NPoM cavity modes. However, since the tip-enhanced PL process is different from the tip-enhanced processes studied in the main text, no direct comparison of the different enhancement factors can be made.

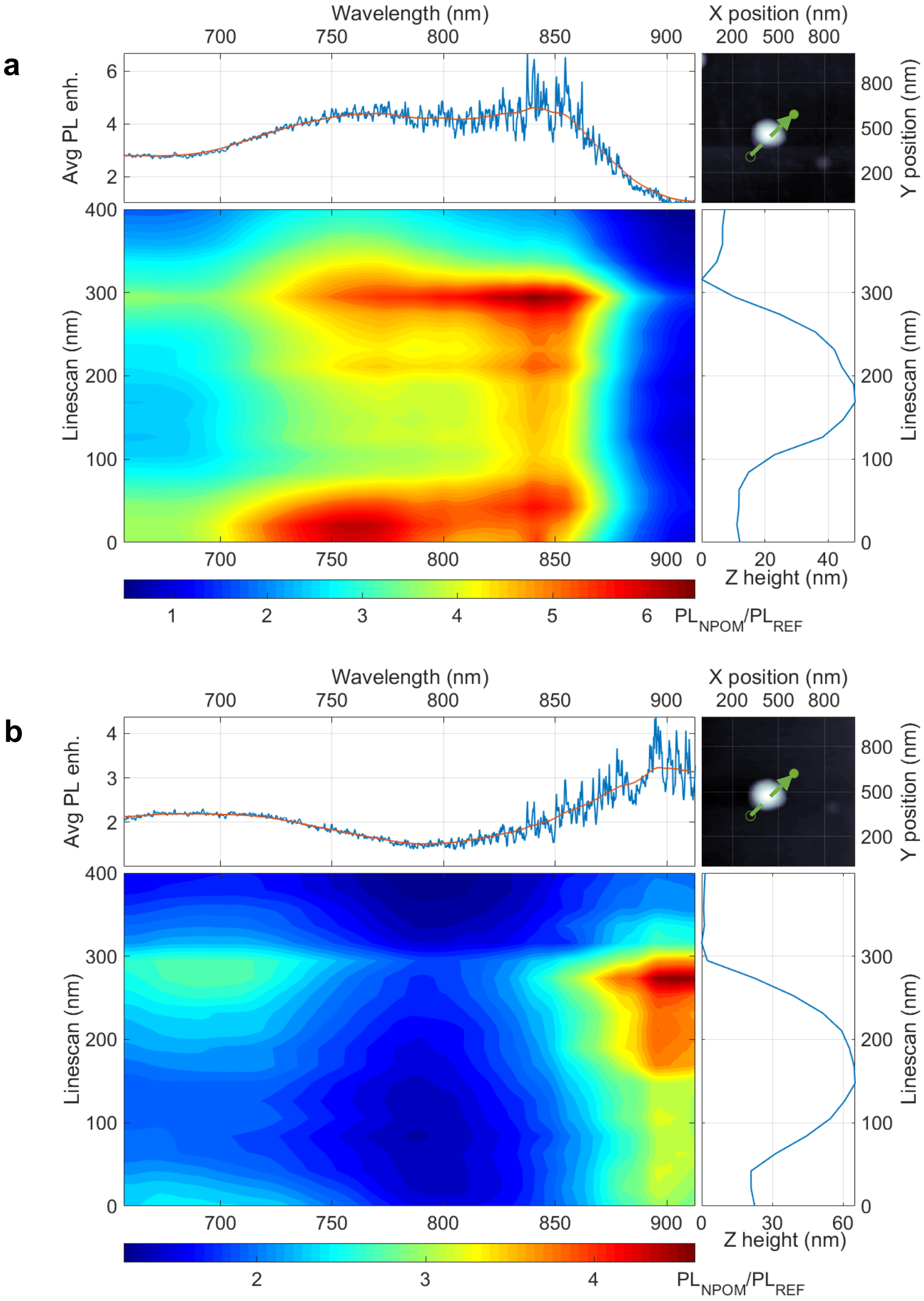

Supplementary Figure S11. **Tip-enhanced photoluminescence (PL).** Spectral variations of PL_{NPoM}/PL_{REF} under illumination at 561 nm (50 μW) as a function of tip position for an isolated gold nanosphere (a) and an isolated gold nanocube (Nanopartz, 80 nm nominal side length) (b) on a BPT functionalized gold film. Panels and experimental parameters are described in Fig. S10.

Supplementary Note 10. Scaling of intensity enhancement factors as a function of tip-NPoM distance

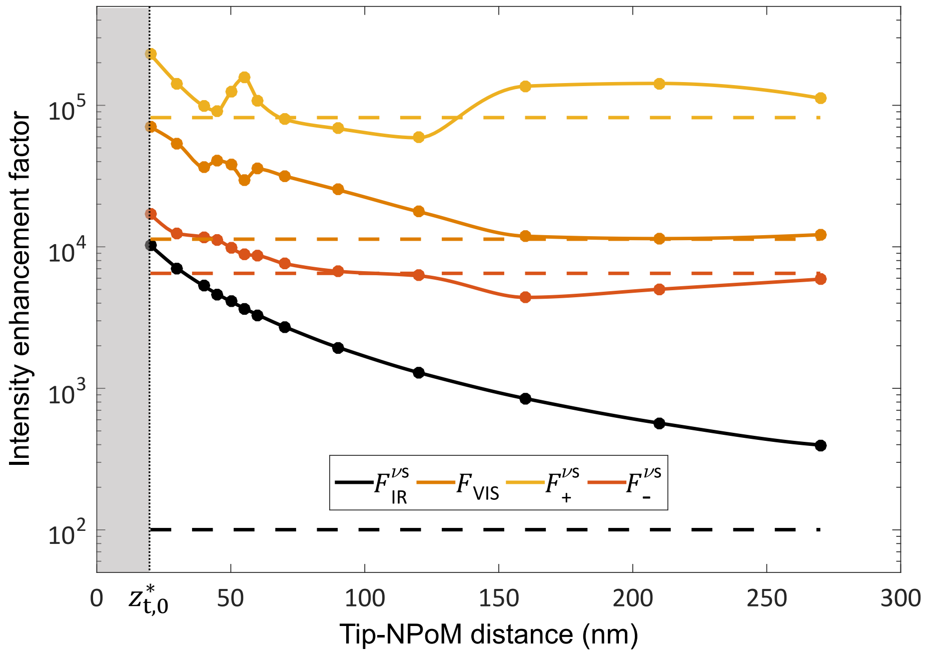

Supplementary Figure S12. **Scaling of intensity enhancement factors as a function of tip-NPoM distance**. Simulated intensity enhancement factors $F^{\nu_s}_{IR}(\vec{r}_{hs})$ (black curve), $F_{VIS}(\vec{r}_{hs})$ (orange), $F^{\nu_s}_{+}(\vec{r}_{hs})$ (yellow), and $F^{\nu_s}_{-}(\vec{r}_{hs})$ (red) at the frequency of the vibrational mode ν_s as a function of vertical tip-NPoM distance. The figure shows that, contrary to the monotonic decrease of the enhancement factor $F^{\nu_s}_{IR}(\vec{r}_{hs})$ for increasing tip-NPoM distance Δz_t in the IR spectral range, the scaling behavior is complex in the visible spectral range (here illustrated for $F_{VIS}(\vec{r}_{hs})$, $F^{\nu_s}_{+}(\vec{r}_{hs})$, and $F^{\nu_s}_{-}(\vec{r}_{hs})$). At large Δz_t, the enhancement factors converge towards the NPoM cavity alone values. Additional non-monotonic features are observed at lower distances (for 40 nm $\lesssim \Delta z_t \lesssim$ 130 nm) and can be attributed to interferences between far-field illumination and tip illumination of the NPoM gap. It is worth noting that even greater intensity enhancement factors are anticipated for tip-NPoM distances smaller than $z^*_{t,0} \simeq$ 20 nm, the effective distance when operating our s-SNOM microscope in contact mode. This region, depicted by a grey area in the figure, remained out of reach for the current version of the microscope.

Associated Content

The data, plotting scripts, and complementary description of the numerical calculations used in this study are openly available in the Zenodo repository: 10.5281/zenodo.15077282.

[S1] McArdle, P., Lahneman, D. J., Biswas, A., Keilmann, F. & Qazilbash, M. M. Near-field infrared nanospectroscopy of surface phonon-polariton resonances. *Physical Review Research* **2**, 023272 (2020).

[S2] Huth, F. *et al.* Resonant Antenna Probes for Tip-Enhanced Infrared Near-Field Microscopy. *Nano Letters* **13**, 1065–1072 (2013).

[S3] Lumdee, C., Yun, B. & Kik, P. G. Gap-Plasmon Enhanced Gold Nanoparticle Photoluminescence. *ACS Photonics* **1**, 1224–1230 (2014).

[S4] Chen, W. *et al.* Intrinsic luminescence blinking from plasmonic nanojunctions. *Nature Communications* **12**, 2731 (2021).

[S5] Tserkezis, C. *et al.* Hybridization of plasmonic antenna and cavity modes: Extreme optics of nanoparticle-on-mirror nanogaps. *Physical Review A* **92**, 053811 (2015).
